# Transcriptome-wide association studies associated with Crohn’s disease: challenges and perspectives

**DOI:** 10.1186/s13578-024-01204-w

**Published:** 2024-02-25

**Authors:** Keyu Jia, Jun Shen

**Affiliations:** 1 Laboratory of Medicine, Baoshan Branch, Ren Ji Hospital, School of Medicine, Nephrology department, Shanghai Jiao Tong University, 1058 Huanzhen Northroad, Shanghai, 200444 China; 2 Division of Gastroenterology and Hepatology, Key Laboratory of Gastroenterology and Hepatology, Ministry of Health, Inflammatory Bowel Research Center, Ren Ji Hospital, School of Medicine, Shanghai Institute of Digestive Disease, Shanghai Jiao Tong University, Shanghai, China; 3 NHC Key Laboratory of Digestive Diseases, Renji Hospital, Shanghai Jiaotong University School of Medicine, Shanghai, China; 4 Division of Gastroenterology and Hepatology, Baoshan Branch, Renji Hospital, School of Medicine, Shanghai Jiao Tong University, Shanghai, China

**Keywords:** Crohn’s disease, Transcriptome-wide association studies, Susceptibility genes, GO functional analysis

## Abstract

Crohn’s disease (CD) is regarded as a lifelong progressive disease affecting all segments of the intestinal tract and multiple organs. Based on genome-wide association studies (GWAS) and gene expression data, transcriptome-wide association studies (TWAS) can help identify susceptibility genes associated with pathogenesis and disease behavior. In this review, we overview seven reported TWASs of CD, summarize their study designs, and discuss the key methods and steps used in TWAS, which affect the prioritization of susceptibility genes. This article summarized the screening of tissue-specific susceptibility genes for CD, and discussed the reported potential pathological mechanisms of overlapping susceptibility genes related to CD in a certain tissue type. We observed that ileal lipid-related metabolism and colonic extracellular vesicles may be involved in the pathogenesis of CD by performing GO pathway enrichment analysis for susceptibility genes. We further pointed the low reproducibility of TWAS associated with CD and discussed the reasons for these issues, strategies for solving them. In the future, more TWAS are needed to be designed into large-scale, unified cohorts, unified analysis pipelines, and fully classified databases of expression trait loci.

**Supplementary Information:**

The online version contains supplementary material available at 10.1186/s13578-024-01204-w.

## Introduction

Genome-wide association studies (GWAS) have been considerably successful in the past decade. From 2005 to 2022, approximately 400,000 single-nucleotide polymorphisms (SNPs) associated with human traits were included in the NHGRI-EBI GWAS Catalog [1, 2]. However, a limitation of GWAS is that approximately 90% of these crucial signals are located in noncoding regions [3]. Transcriptome-wide association study (TWAS) is a bioinformatic approach that integrates large-scale GWAS, uses expression quantitative trait loci (eQTL) datasets to predict gene expression levels, and attempts to identify disease-related genes and verify associations of interest. This is important for exploring specimens that are not easily collected and phenotypes rarely collected with genetic data. TWAS can assess the association between variation in gene expression levels and phenotypic variation based on different population genotype and tissue-specific gene expression data—an additional analytical approach to GWAS data—and may be used to screen candidate pathogenic genes.

Crohn’s disease (CD), a type of inflammatory bowel diseases (IBD), is a lifelong progressive disease with a tendency of symptoms to flare up or subside as the condition alternates between active and remission periods [4]. All segments of the gastrointestinal tract can be affected. The Montreal classification introduced subgroups of CD by considering the location of the disease, age of onset, and phenotype [5]. In recent decades, many researchers have believed that the pathogenesis of CD is an irrepressible immune response to luminal bacterial antigens. Immune cells participate in this process when infiltrating the gut of CD patients. Therefore, the onset of CD involves the immune system, segments of the digestive tract, intestinal tract contents, and multiple organs, which experience complications.

Over the past decades, GWAS have identified over 240 IBD susceptibility genes or loci outside the human leukocyte antigen region, and 37 of these genes are specific for CD [6, 7]. However, many of the polymorphic sites associated with CD are located in non-transcribed regions and do not cause amino acid substitutions or functional mutations, nor do they exhibit disease susceptibility. For instance, the GWAS results of Japanese patients with CD showed that among the 11 susceptibility gene loci associated with CD, only rs76418789 located in *IL23R* had an amino acid substitution [8]. The other 10 polymorphic sites may be located in these disease-susceptible regions, thereby affecting the expression of nearby genes and participating in the occurrence and development of CD [9]. However, the function of these SNPs in the occurrence and development of CD remains unclear. Thus, TWAS must be conducted to provide a biological context for interpreting disease risk loci by nominating candidate susceptibility genes not only in GWAS risk regions but also in other regions of potential that cannot be detected by current GWAS.

Therefore, we aim to provide an overview of previous TWAS on CD and summarize the databases and methods used in these studies. This review also discusses the overlapped susceptibility genes in different tissues and the potential pathway involved in CD by GO functional analysis, which may provide clues to explore the pathogenesis, diagnosis, and classification of CD.

## Heterogeneity across TWASs of CD

**Fig. 1:**
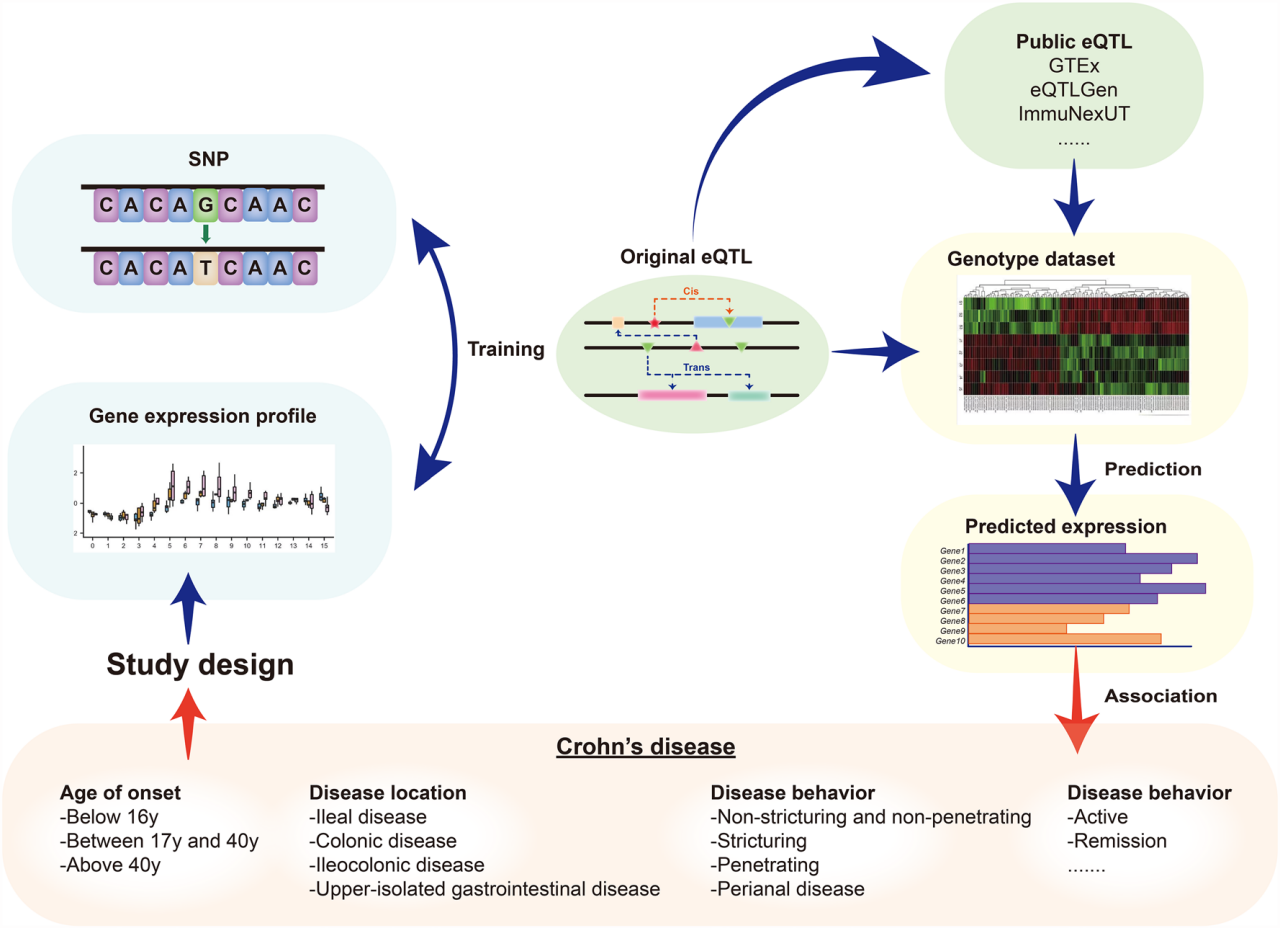
Workflow of TWAS in CD. The initial stage of TWAS design should consider characteristics of CD such as disease behavior, disease location, and disease status, and select GWAS datasets and eQTL populations with specific CD subtypes to ensure homogeneity within studies. TWAS could choose the public eQTL database or establish an original eQTL. The large-scale genotype dataset is used to predict expression data by eQTL to associated with CD related outcomes. *CD* Crohn’s disease, *TWAS* transcriptome-wide association studies, *SNP* single nucleotide polymorphism, *eQTL* expression quantitative trait loci

There were seven TWASs for CD overviewed in this review with details in Table 1. Four of these studies conducted TWA in single tissue types of Japanese [9], Korean [10], American [11], and British [12] populations. In particular, the Japanese study also used a cross-tissue eQTL database to explore susceptibility genes based on genotypic data from the Japanese population [9]. The other three studies used cross-tissue and multi-country eQTL designs [13–15]. The selection of GWAS datasets, eQTLs, sample sizes, tissue types, and screening criteria varied among previous TWAS (Fig. 1).

**Table 1:** Summary of the basic information of TWAS studies in CD

Year	GWAS data					eQTL	Methods	Associated tissue type	Association screening criteria
	Country	Database	N	Country	Tissue type of RNA-seq	N			
Diez-Obrero et.al. [15]	Europe	IBD GWASs of European ancestries	IBD: 25,042 HC: 34,915	Spain (BarcUVa-Seq) Europe (CEDAR)	Ascending colon	HC:138	S-PrediXcan	Ascending colon, Transverse colon, Descending colon	P _Bonferroni_ < 0.05
					Transverse colon	HC:143			
					Descending colon	HC:164			
Uellendahl-Werth et. Al. [13]	15 countries cross Europe, North America, Australia	Ten case–control GWAS datasets	CD: 21,771 HC: 41,206	US (GTEx), Sweden (STARNET), UK (BLUEPRINT)	Small intestine	non‐IBD 77	UTMOST	Small intestine, Sigmoid colon, Transverse colon, Whole blood, CD14 + Monocytes, CD16 + Neutrophils, Naïve T cell	P _Bonferroni_ < 0.05
					Sigmoid colon	non‐IBD 124			
					Transverse colon	non‐IBD 169			
					Whole blood	non‐IBD:338			
					CD14 + Monocytes, CD16 + Neutrophils, Naïve T cell	non‐IBD 338			
Kakuta et.al. [9]	Japan	CD GWASs of Japanese	CD: 713 HC: 2063	Japan	Intestines	active CD: 15 active UC: 5	GWAS-eQTL analysis	Effector memory T cells from inflammation sites	Susceptibility: P _FDR_ < 0.05 Candidate: P _FDR_ < 0.1
				US (GTEx)	Sigmoid colon	HC: 203	FUSION	Sigmoid colon, Transverse colon, Small Intestine, Whole blood, EBV transformed lymphocytes	
					Transverse colon	HC: 246			
					Small Intestine	HC: 122			
					Whole blood	HC: 369			
					EBV transformed lymphocytes	HC: 117			
Jung et.al. [10]	Korea	CD GWAS of Korean	CD: 899 HC: 3805	Korea	Peripheral blood	CD:101	FUSION	Whole blood	P _Bonferroni_ < 0.05
Gettler et.al. [11]	Cross countries	15 GWAS of CD and/or UC	CD: 6299 HC: 15,148	America (GEO)	Terminal ileum	CD: 213, UC: 50, Unspecified IBD:4, HC: 35	COLOC	Terminal ileum	P _FDR_ < 0.1
Dai et.al. [12]	Europe	IIIBDGC	CD: 5956 HC: 21,770	British	Whole blood	CD: 24 HC: 23	TESA MetaXcan	Terminal ileum Whole blood Spleen	FC ≥ 1.5 or ≤ 0.67 P _Pascal_ < 0.05
Cheng et. al. [14]	Europe	IBD GWAS of European ancestries	CD: 18,405 UC: 14,308 HC: 34,241	Cross countries (GEO)	Intestinal	IBD: 134, non‐IBD: 134	FUSION	Sigmoid colon, Transverse colon, Whole blood/ Peripheral blood, Small Intestine terminal ileum EBV transformed lymphocytes	P-value < 0.05 & FC > 1.5
					Whole blood	IBD: 75, HC:12			
					Peripheral blood	HC:42, UC:26, CD:59			

*CD* Crohn’s disease, *UC* ulcerative colitis, *IBD* inflammatory bowel disease, *HC* healthy control, *GWAS* genome-wide association studies, *TWAS* transcriptome-wide association studies, *eQTL* expression quantitative trait loci; *PGC* Psychiatric Genomics Consortium, *IIIBDGC* International Inflammatory Bowel Disease Genetics Consortium, *GEO* Gene Expression Omnibus database, *BarcUVa-Seq* The University of Barcelona and University of Virginia RNA sequencing project; *CEDAR* correlated expression and disease association research, *STARNET* Stockholm-Tartu Atherosclerosis Reverse Networks Engineering Task study, *S-PrediXcan* Summary-PrediXcan, *FC* fold change

### Selection of genotype data

Large-scale genotype data provide fundamental material for TWAS. Many multi-country GWASs reported to be associated with CD provide investigators with a variety of options for data selection [16]. As the genetic structure of disease-causing mutations varies in different populations, the effect sizes and risk prediction scores derived for SNPs in one population may not be directly generalizable to other populations [17, 18]. CD has a great ancestral dependency by comparing the GWAS data from East Asian and European ancestries [19]. As yet, most of the available genetic information is based on data from populations of European ancestry [20–22]. Key variants that are low in frequency or absent entirely in European populations are likely to be missed when studied in other populations, especially if the variant is ethnic-specific, leaving additional blind spots for future studies [23, 24]. Therefore, considering the study population during the study design stage, caution should be exercised in genotype dataset selection. Most of the 7 TWAS selected consistent races to establish this relationship. For example, the Japanese and Korean TWAS selected genotype and expression populations from their countries. Dai et al. chose genotyped data of European ancestry as the GWAS population and 24 CD patients and 23 healthy controls from the IBD–BIOM inception cohort from UK [12, 25]. Gettler et al. used both the genotype and expression populations of the Childhood CD Study derived from the RISK cohort [11]. However, the selection of a consistent population race may limit the sample size of the study.

### Selection of eQTL data

eQTL datasets are constructed using statistical models based on genotype and tissue expression data from the same population, which is an effective tool for fine-mapping GWAS that identifies SNPs associated with complex phenotypic traits and can be used to improve the heritability explained by identifiable genetic factors and to better understand the biological basis of complex traits [26].

Previous TWAS exhibited acute instability when choosing various eQTL datasets based on different tissue types. Since eQTL limited the specific tissue to find differentially expressed genes, overlapping the same GWAS database with eQTLs of different tissues could result in different results of TWAS.

eQTLs are race-specific in several aspects. (1) For a specific phenotype, causal genes might be distinct across ethnicities. (2) Many polymorphisms were rare in some races but common in others, which could ignore some associations between SNP and expression in the application. (3) The degree of association between a specific SNP and the expression level in one race might also differ from that in others. Owing to these diversities, when utilizing the same GWAS dataset, substantial discrepancies exist in the conclusions drawn among different studies conducted in different countries. As can be seen in Additional file 1: data S1, for the same TWAS methods, it tends to screen out more genes with a consistent ethnicity of GWAS population and eQTL population. For example, for whole blood, the Japanese study used GTEx (eQTL from the United States) to screen out only 1 gene, and the Korean study used their own eQTL to screen out a total of 21 genes, which also suggested that eQTLs are race-specific and the consistent ethnicity of GWAS and eQTL populations may increase the accuracy of expression prediction.

Whether eQTLs are disease-specific is uncertain [27]. Because epigenetic modifications differ according to the disease state [28], the relationship between expression mapping and genotype could also be affected. In our review, most TWAS combined the expression data of healthy individuals and CD patients into the same eQTL establishment. Uniquely, the Japanese study selected data from CD and UC patients to construct its own eQTL dataset of IBD [9], which may interfere with the homogeneity of eQTLs in expression prediction and further association analysis between CD patients and healthy controls though reduce the test power concomitantly. The relationship between eQTL and GWAS associations at the same locus could be unpredictable for different disease types. A previous study of non-alcoholic fatty liver disease used the disease-specific eQTL to pinpoint individuals that harbor specific genotypes more or less susceptible to the disease [29]. Thus, using disease-specific eQTL to establish the relationship in patients is worth investigating.

The resolution is low and unreliable with a sample size of 100 [27], though the sample size of the original eQTLs in most CD TWASs did not exceed 50 pairs. Various public eQTL databases are available to explore CD, including the Genotype-Tissue Expression (GTEx, https://www.genome.gov/Funded-Programs-Projects/Genotype-Tissue-Expression-Project), eQTLGen (https://www.eqtlgen.org/phase1.html), and the Blood eQTL browsers (http://genenetwork.nl/bloodeqtbrowser/). Although GTEx has been in development for 10 years, it is still worth using because its data is relatively stable and still update yet with a wide range of tissue types and sample size. As the largest eQTL database, GTEx data included genotype data from 714 donors and 11,688 RNA-seq data from 53 tissue sites and two cell lines, with sufficient assay power to establish eQTLs in 48 tissue types/sites. Although the database only included the data of a healthy population, it could be combined with the expression database of CD patients for further analysis. As GTEx included data/patients solely from the United States, its utility is limited for other countries’ populations. eQTLGen included 37 datasets from 31,684 individuals, including cis-eQTL, trans-eQTL, eQTS, and single-cell eQTLGen Consortium; however, only blood samples were used [30].The Blood eQTL Browser, which has 5311 individuals’ data, also included only blood samples [31], which limited the exploration of other tissue types more likely to be causally related to CD. Therefore, a database with more comprehensive and specific classifications across tissue types, diseases, and ethnicities is warranted to facilitate the use of multiple disease-targeting tissue types in future large-scale eQTL studies and to provide a unified platform for mining more robust associations in next-generation studies.

### Selection of software or methods of TWAS

Integrating GWAS and expression data in TWAS could performed by various tools, including PrediXcan for individual-level GWAS data, Fusion and S-PrediXcan for summary-level GWAS data, closely related methods, including SMR and HEIDI, based on Mendelian randomization (MR), and GWAS–eQTL colocalization methods, including Sherlock, coloc, QTLMatch, eCaviar, enloc, and RTC, for detecting genes whose expression regulated by the same GWAS hit [32, 33].

Of the seven CD TWAS, five TWAS used commonly used methods such as Fusion, PrediXcan or MetaXcan to integrate GWAS and expression data. MetaXcan is an expanded method calculating the results PrediXcan without using individual data [33].

Besides, Uellendahl-Werth et.al conducted cross-tissue TWAS associated with gut-brain-axis performed by UTMOST (multivariate-response penalized regression models) to predict cross-tissue gene expression [13]. And they observed that UTMOST could get more moderate associations and effectively select predictive cis eQTL variants compared with S-PrediXcan (logistic regression model) and FUSION (Bayesian linear mixed model) [13]. When comparing across studies, the genes screened by ULMOST and MetaXcan rarely overlap with those screened by traditional methods (Additional file 1: Data S1), which suggests that the methods bias was not the main result responsible for the poor reproducibility. Since Uellendahl-Werth et al. did not present the associated genes screened by S-PrediXcan and FUSION in their results, whether method bias contributed to the low repetition rate is uncertain.

Gettler et al. [11] used the coloc R package, which, as Wainberg et al. previously mentioned, is vulnerable because co-regulation bias makes it difficult to distinguish causality based on GWAS and expression data [32, 34]. Hukku et al. compared PrediXcan and GWAS–eQTL colocalization methods and found that the GWAS–eQTL colocalizations may have a higher specificity and limited sensitivity, and PrediXcan could be possible to report more results with difficult in biological interpreting [35]. Therefore, caution should be used when interpreting TWAS test results derived from controversial methods, as some of them may simply be false hits. [32].

## Heterogeneity of various tissue types selected in eQTL

**Fig. 2:**
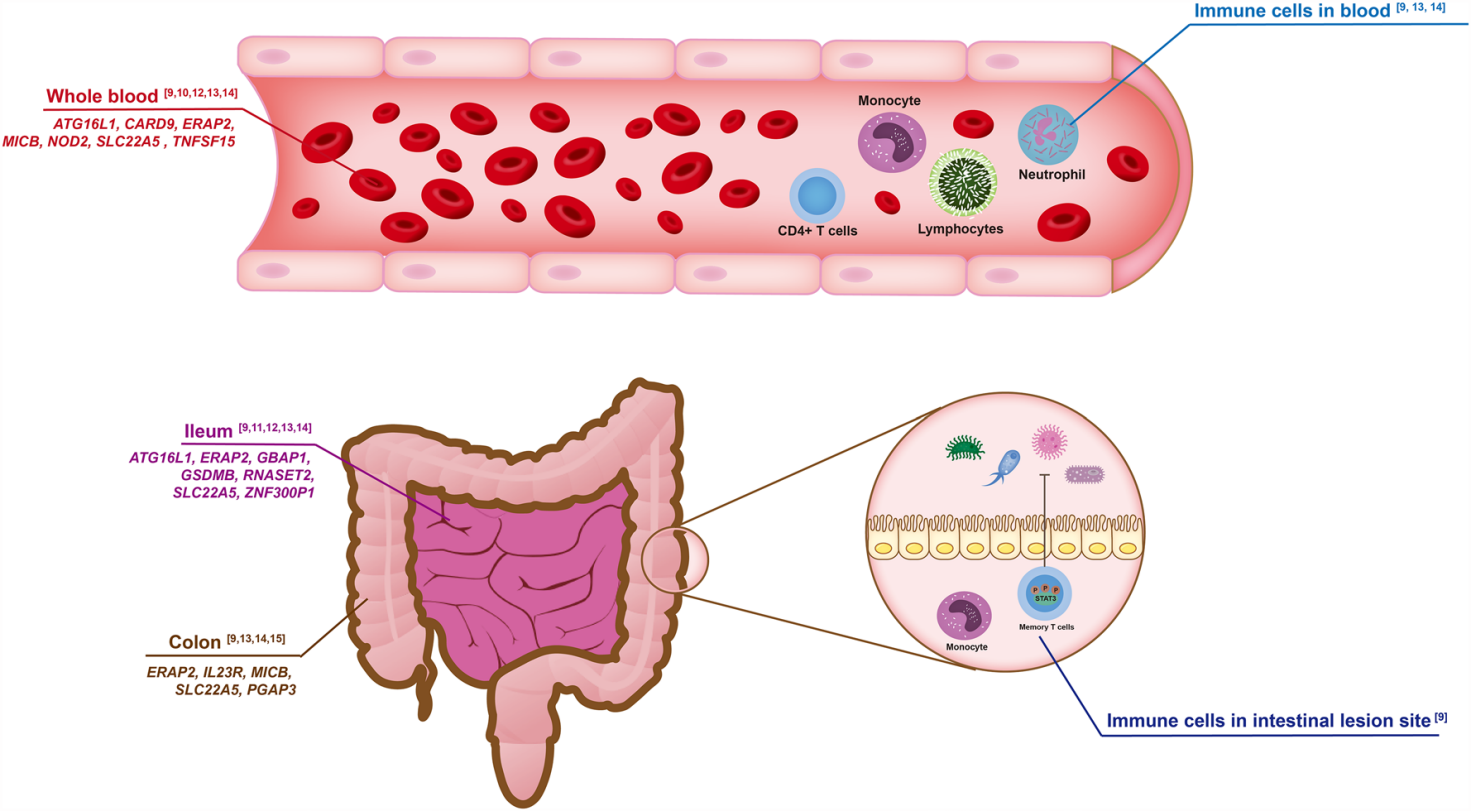
Summary of susceptibility genes associated with CD in different tissue types founding in TWAS. The bold gene names were the susceptibility genes at least found in two TWASs

All segments of the digestive tract, including the systemic immune inflammatory response, could be involved in CD. Previous reviews have summarized the differences in the epidemiology, genetics, histology, microbiology, and immunology of ileal and colic celiac disease, suggesting that CD at different lesion sites should be regarded as distinct subtypes [36, 37]. Each segment of the digestive tract, immune cells, and other complex organs can be used as follow-up tissues to explore the unique pathology and etiology of CD. To date, only blood, immune cells, colon tissue, and ileum tissue have been reported in previous TWAS for CD. In this section, several biospecimen-related issues of utmost concern are discussed. Susceptibility genes associated with CD reported in TWASs in different tissue type were took union set and summarized in Additional file 2 Data S2. The overlapped susceptibility genes between TWASs are visualized in Fig. 2.

**Fig. 3:**
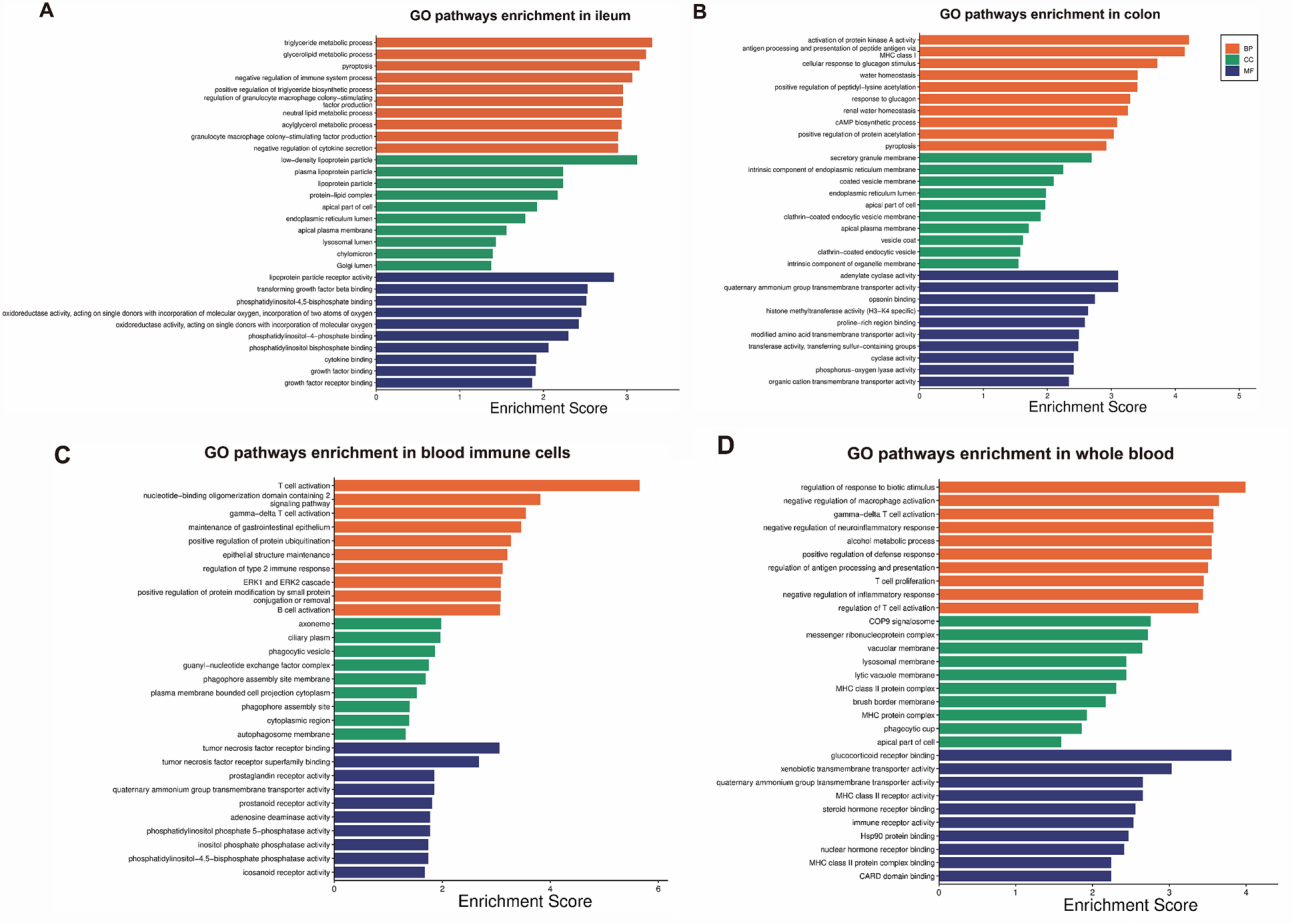
The top 10 results obtained from GO functional analyses of all susceptibility genes associated with CD in each tissue type. *BP* biological process, *CC* cellular component, *MF* molecular function

In this review, GO functional analyses of all susceptibility genes associated with CD in each tissue type (Shown in Additional file 2: Data S2) were conducted by “clusterProfiler” and “pathview” packages of R software [38, 39]. Since the logFC of these associated genes were not available, the GO function enrichment analysis was roughly conducted with a random assignment of foldchange (1 or -1), which can only suggest relevant functional enrichment and cannot indicate up-regulation or down-regulation. The top 10 results for significance (P value < 0.05) of Cellular Component (CC), Molecular Function (MF), and Biological Process (BP) are shown in Fig. 3. And the total significant results were shown in Additional file 3 data S3.

### Digestive tissue

Clinical inflammation in CD can infect the entire gastrointestinal tract from the mouth to the anus [40]. The intestinal epithelium is a single layer of columnar epithelium that produces mucus and antimicrobial factors and can establish a buffer zone between the luminal contents and itself [4]. Destruction of the intestinal epithelium may cause bacterial invasion and lead to the occurrence of CD. Thus, in CD studies, epithelial lesion tissue is commonly seen as the top-priority casual biospecimen [41].

A comparative study observed that 19 hub genes were differentially expressed between the colon and ileum [42], suggesting that the tissues of different intestinal segments should be explored separately. Taking Th17 cells—identified in the pathogenesis of IBD—as an example, the genes related to Th17 pathways involved in CD were inconsistent between the colon and ileum, and the regulation (up or down) of mRNA expression levels of these genes in the colon and ileum lesion tissue from CD patients were also inconsistent [43–49]. These data suggest that gene expression patterns are significantly distinct at different intestinal sites.

#### Ileum

The ileum, filled with abundant bile and digestive juice, is a relatively germ-free environment. A recent study found a trace amount of microbiota in the ileum, which differed radically from colonic microbiota [50]. A cultivable bacterial density of approximately 104 CFU/mL in the ileum was also much less than the 1011–1012 CFU/mL in the colon [51]. This might indicate that the ileum and colon are situated in different external environments, which could stimulate differential gene expression.

Five TWASs selected ileum as etiological tissue of CD. *ATG16L1* [11, 13], *ERAP2* [11–13], *GBAP1* [11, 12], *GSDMB* [11, 12], *RNASET2* [11, 12], *SLC22A5* [11, 12], and *ZNF300P1* [11, 12, 14] in the ileum were susceptibility genes overlapped between at least two TWASs.

*ATG16L1* has been reported as an autophagy-related gene [6] and a major risk polymorphism in CD [52]. In a genotypic study in a U.K. population, the strongest association was observed for the *ATG16L1* risk variant with ileal disease [53]. And William J. Sandborn commented that *ATG16L1* genotype is associated with response to anti-TNF therapy. The *ATG16L1* mutation results in altered function and survival of highly specialized secretory epithelial Paneth cells located in small intestinal crypts, resulting in decreased secretion of antimicrobial proteins [54, 55]. The CD-associated *ATG16L1* mutation observed in animals is associated with low clearance of *Yersinia enterocolitica* and adherent-invasive *Escherichia coli*, which further increase the production of inflammatory cytokines [56, 57]. And several studies have reported that the presence of the adherent-invasive *E. coli* abnormally colonize the ileal mucosa of CD patients [58]. Thus, the *ATG16L1* mutation may be involved in the pathological process of CD by causing intestinal barrier disruption. The expression of ATG16L1 could be targeting regulated by a variety of micro-RNA, including miR-106b found in intestinal epithelial HCT116 cells, and four microRNA (miR-106a [59], miR-874 [60], miR-410 [61], and miR-223 [62]) found in cancer or other inflammation disease, which may provide pharmaceutical strategies for developing small molecule drugs for CD.

*ERAP2* shows limited polymorphism coding for Lys392Asn change, which affects the activity of aminopeptidases [63]. *ERAP2* forms a repertoire of ligands for HLA class I, involving in the processing of MHC-I ligands antigen presentation and the antigenic response of infection [64], which associated with various inflammation diseases, such as IBD, ankylosing spondylitis, birdshot chorioretinopathy, Behcet’s disease and psoriasis [65–67]. In this review, *ERAP2* is also observed an overlapping susceptibility gene in colon and blood, which has potential application in clinical screening and diagnosis of CD. Accumulating evidence suggests that *ERAP2* is tractable targets for the regulation of immune responses [68]. In pancreatic cancer cells, gemcitabine could increase the mRNA and protein levels of ERAP2 [69]. However, no drugs targeted ERAP2 in CD yet.

*GBAP1* is a pseudogene for the glucocerebrosidase (*GBA*) gene encodes for the enzyme glucocerebrosidase. Previous studies have demonstrated that *GBAP1* can act as a competitive endogenous RNA to competitively bind with microRNAs in gastric cancer [70, 85] and Parkinson's disease [71] through functional prediction, thereby promoting the expression of *GBA*. However, the role of glucocerebrosidase in CD has not been reported.

*GSDMB*, a member of the Gasdermins family, was originally known for its role in pyroptosis [72], and most prevalently expressed in gastrointestinal-associated organs, including stomach, small intestine and colon [73]. Studies have found that the expression of GSDMB is increased in the inflammatory mucosa of ileum and colon of CD patients, and the related genes are enriched in cell proliferation, migration, and adhesion other than of pyroptosis [74]. As an inducer of GSDMB, methotrexate could induce upregulation of IEC-derived GSDMB-FL and translocation to the plasma membrane, but not lytic cell death in undifferentiated HT-29 cells. And the development of methotrexate in CD tarfeting GSDMB has entered phase III clinical trials (NCT00132899, Table 2).

**Table 2:** Targeted drugs had been developed of overlapped genes

Genes	Target	Regulatory factor/drug	Disease	Developed process	Clinical trials. gov identifier
*GSDMB*	GSDMB	Methotrexate	CD	A Phase III randomized, placebo-controlled, double-blind	NCT00132899
*IL23R*	IL23 p19	Brazikumab	CD	The Phase 3 trial was terminated	NCT03961815
		Risankizumab	CD	In phase 3, randomized, placebo-controlled, double-blind trial	NCT06063967
		Mirikizumab	CD	In Phase 3 open-label trial	NCT04232553
		Guselkumab	CD	In Phase 3 randomized, placebo-controlled trial	NCT05347095
	IL23R	JNJ-67864238	CD	Study terminated early as futility criteria met	NCT04102111
*TNFSF15*	TNFSF15	PF-06480605	UC	Phase 2a single-arm trial	NCT02840721

*CD* Crohn’s disease, *UC* ulcerative colitis

*RNASET2* is the only human member of the Rh/T2/S family of acidic hydrolases [75]. An eQTL analysis observed an association between decreased *RNASET2* and *TNFSF15*-mediated IFN-γ production, a key mediator of mucosal inflammation [76]. The circulating RNASET2 protein levels was decreased in CD patients compared with healthy control [75]. In cancer studies, *RNASET2* has been found to be involved in recruitment, activation, and polarization of monocytes and macrophages [77, 78]. However, the role of RNASET2 in CD needs to be further investigated.

*SLC22A5* code organic cation transporters (OCTN2), which was widely expressed and also the susceptibility genes observed in colon and blood (can be seen below). OCTN2 is mainly localized at the brush-border of apical membranes of intestinal epithelial cells and has a high transport capacity of L-carnitine in the small intestine, which is vital for β-oxidation of long-chain fatty acids in the mitochondria [79]. Several studies have observed the expression of OCTN2 downregulated in inflamed sites compared with non-inflamed sites both in patient intestinal tissue and mice model [80, 81]. And the PPARα/γ may act as transcription factors in the expression of OCTN2 and further regulate inflammatory response [80]. OCTN2 also could transports drugs, such as TEA, ipratropium, prednisolone, and beta-lactam antibiotics [82–84].

*ZNF300P1* encode a long intergenic noncoding RNA, suggesting its primary function may be to regulate expression of other genes [85]. *ZNF300P1* was found upregulated in ileum, rather than in colon or whole blood [86]. Besides, ZN*F300P1* may alter tissue-specific expression of TNF and a range of additional genes previously implicated in colitis and/or autophagy. Besides, *ZNF300P1* is reported to regulate polarity, proliferation, migration, and adhesion in ovarian epithelial cells [87], suggesting that it may similarly participant in intestinal epithelial functions.

The pathways of susceptibility genes associated with CD in ileum were enriched in lipid-related metabolism (Fig. 3A). Previous observational studies have reported a distinct lipid profile in CD patients compared with healthy population [88, 89]. And growing evidence showed emulsifying omiga-3 fatty acids maybe a potential supplementary in maintaining remission of CD patients [90, 91]. An epidemiological study observed that lower total cholesterol levels, LDL-C, and HDL-C were associated with higher incidence of CD, but not UC [92]. Coincidentally, another study also observed that more lipid components significantly changed in CD patients than in UC patients compared with healthy population [93]. Considering that ileal lesions are present only in CD, the different association of lipid metabolism with these two types of IBD may be due to the location of the lesion in the ileum. However, A shotgun lipidomics study of noninflammatory ileal biopsy tissue identified only phosphatidylinositol 16:0/18:1 was different between healthy controls and CD patients, although the sample size was small [89]. Additional future exploration will be necessary to confirm this observation.

#### Colon

Unlike the ileum, the colon has a substantial bacterial load, which plays a crucial role in regulating gut health. Changes in the abundance of specific bacteria have been used as biomarkers for screening gastrointestinal disorders, including IBD, irritable bowel syndrome, adenomatous colonic polyps, and colorectal cancer. Changes in the abundance of some bacteria have been used as biomarkers to screen for IBD and other gastrointestinal diseases [94–96]. A TWAS conducted in the gut microbiota has detected multiple tissue-specific candidate genes in the sigmoid and transverse colon, respectively, such as TOB2P1 for Enterococcaceae in sigmoid colon, WDR6 for Coprococcus in sigmoid colon, and KCNIP3 for Veillonellaceae in transverse colon [97]. An association study using bioinformatic analysis in colorectal cancer also observed two overlapping pathways, the bile secretion and steroid hormone biosynthesis pathways, enriched by operational taxonomic units (OTUs) and gene expression patterns in colon tissue, respectively [98]. These results indicate a close cross-talk between the intestinal microbiota and the colon transcriptome. Thus, the results of colon TWAS could identify potential genes stimulated by the microbiota and provide hints to explore the colon-specific pathology of CD.

Four TWASs included colon as casual tissue, and each study included at least 2 segments of colon. In several studies, susceptibility genes observed in the sigmoid and transverse colon almost overlap. In Japanese population, among five genes observed in colon, four genes, including *ERV3*-1, *NPIPB9*, *ZNF713*, and *WDR31* overlap between sigmoid and transverse colon [9]. However, none of these genes overlap with the results of other reports. For the cross-tissue TWAS, Uellendahl et al. found that 18 genes were differentially expressed in the sigmoid colon, and 31 genes were differentially expressed in the transverse colon [13]. Seven genes were overlapped between two segments of colon. In European ancestries, Cheng et al. found that *ZNF300P1* and *MICB* were significantly differentially expressed in both the sigmoid and transverse colon among the 3 susceptibility genes in colon [14]. In a meta-analysis, Virginia et al. conducted TWAS in three colon segments, including the ascending, transverse, and descending colon. And three colon segments had 11 overlapping genes, including *SLC22A5, GSDMB, ENTR1, ERAP2, C4A, FUT2, UBA7, GSDMA, FLRT3, RBM6,* and *HLA-C* [15]. In the comparison between Uellendahl’s and Virginia’s studies, *ERAP2* and *IL23R* in the transverse colon were observed in both. None of these susceptibility genes was replicated across studies in the sigmoid colon, because two of these studies found a relatively small number of genes (Additional file 2: data S2) [9, 14].

Among all susceptibility genes reported in colon regardless of which segments, *ERAP2* [13, 15], Interleukin-23 receptor (*IL23R*) [13, 15], Major histocompatibility complex class I chain- related gene B (*MICB*) [14, 15], Post-GPI attachment to the proteins 3 *(PGAP3)* [13, 15], and *SLC22A5* [14, 15] were overlapped between TWASs. *ERAP2* and *SLC22A5* were also overlapped with ileum discussed above.

*IL23R* is one of popular genes affects disease susceptibility and highly expressed on cell membrane of memory T cells and other immune cells, such as natural killer cells, monocytes, and dendritic cells [99]. *IL23R* interacts with IL-23, regulating the of immune activity and against infection by bacteria and viruses [99]. And the functional *IL23R* pathway polymorphisms play a role in modulating neonatal development of intestinal tolerance and bacterial colonization [100]. There were several of humanized monoclonal IgG, including Brazikumab, Risankizumab and Mirikizumab, could binds p19 of IL23 has entered clinical trial, and most of them has enter phase 3 clinical trials (Table 2). Due to the presence of protective or disease-associated variants in IL23R and related genes, only one locally acting oral peptide (JNJ-67864238) directly antagonizing IL-23R was found but was recently terminated after meeting criteria for futility [NCT04102111] [101].

*MICB* almost exclusively expressed in the intestinal epithelium [102]. *MICB* was reported in many human cancers via immune evasion [103–105]. And the immune cells, including natural killer (NK) cells and T cells, involved in *MICB* were also connected with CD. However, the functions of *MICB* in CD were still lack of evidence. In addition, *MICB* has only been reported as a CD susceptibility gene in whole blood, but not in blood immune cells, which needs further study.

*PGAP3* is ubiquitously expressed and code a Glycosylphosphatidylinositol (GPI)-specific phospholipase involving in lipid remodeling of GPI-anchored proteins[106]. The function of PGAP3 was most reported in brain morphogenesis and mental development [107, 108]. However, the mechanism of PGAP3 in CD was still under studied.

To be noted, the susceptibility genes associated with CD in colon enriched in the cell component of vesicle membrane (Fig. 3B), including exosomes, microvesicles and apoptotic bodies from endosomes, plasma membrane, plasma membrane/endoplasmic reticulum, respectively [109]. And the vesicle may be related with bacteria–host communication, which may involve in internalization of bacterial extracellular vesicles of epithelial cells [110]. Endocytic routes of intestinal epithelial cells, including macropinocytosis, clathrin-mediated endocytosis and lipid raft-mediated processes, may involve in CD pathogenesis [109]. Furthermore, extracellular vesicle (EV), mainly secreted by immune cells and intestinal epithelial cells, could package double-strand DNA (dsDNA), activating the STING pathway to provoke inflammatory responses [111]. Increasing evidence found EVs containing nucleotides have the potential to be biomarkers for the diagnosis of UC or general IBD [112–115]. And EVs may have therapeutic value for IBD [116]. However, the mechanism of EVs in the pathogenesis of CD remains to be further explored.

### Immune cells

The clinical symptoms of CD, including fever, diarrhea, and abdominal pain, mainly depend on the site of inflammation [117]. Various combinations of immune cell types and their locations may also indicate discrepant bio-metabolic pathways and pathogeneses.

#### Intestinal immune cells

CD development involves a combination of environmental, microbial, and immune-mediated factors in individuals with susceptibility gene mutations [118]. Population studies have reported the highest incidence of CD activity in areas with high bacterial counts (colon) and relative retention of fecal material (terminal ileum and rectum) [119, 120]. Once bacteria destroy a single layer of columnar epithelium of the gut, the mucus and antimicrobial factors produced by the intestinal epithelium cannot defend against bacterial invasion [121], and the immune response occurs first in intestinal tissue. Thus, immune cells in the intestine could reflect the ultimate origin of CD.

At present, only Japanese studies have performed TWAS on immune cells in intestinal tissues using their own genome-wide and transcriptome data, and only *TNXA* was found to be significantly differentially expressed in CD4 + effector memory T cells (TEM cells) derived from lamina propria mononuclear cells (LPMCs) in the inflammatory sites of intestinal tissues [9]. Epstein–Barr virus (ERV) 3–1 in EBV-transformed lymphocytes was identified as a susceptibility gene for CD in Japanese patients using the GTEx database [9]. However, this gene was not screened in the populations of Western countries [14]. Owing to the small sample size, the study also broadened the significance level and defined *RAP1A* as a candidate gene associated with CD (FDR < 0.10) [9].

#### Immune cell in blood

As the disease progresses, lesion locations may change or increase, and the risk of complicated diseases, such as rectal disease and perianal lesions, also increases. The metabolites of the microbiota associated with CD participate in immune progress, which can provoke the autoimmune response of the whole body [122]. Therefore, the genetics of immune cells in the circulatory system could also reflect the pathogenesis of CD.

There were three studies include blood immune cells as targeted tissue type. *ATG16L1*, *NOD2*, *ZGLP1*, *BRD7*, *CISD1*, and *SNX20* were significantly related to CD in multiple immune cells, including naïve CD4 + T cells, CD14 + monocytes, and CD16 + neutrophils, in the same TWAS [13]. No gene was found to be significantly associated with CD using gene expression data from the Gene Expression Omnibus database to explore related genes in EBV-transformed lymphocytes, [14]. Because the diversity of immune cell types varies widely, no overlapped susceptibility gene in blood immune cells were observed cross different TWASs.

Susceptibility genes in blood immune cell reported in TWASs are involved in the activation of immune cells and the maintenance of gastrointestinal epithelium (Fig. 3C), suggesting that immune cells may tend to function in the gut, where they may be more susceptible to CD.

### Blood

Whole blood is a heterogeneous tissue that includes a variety of immune cells, including lymphocytes, neutrophils, monocytes, and macrophages, with unique and disease-related roles in CD pathology. Extensive studies of whole blood or lymphoblasts are often used to maximize test power; however, they are mechanistically less relevant to disease. Owing to the relatively low cost of DNA and RNA extraction from whole blood, choosing whole blood for early exploration with a large sample size is feasible.

In a multi-tissue analysis using the GTEx in humans, compared with other tissue types, whole blood exhibited the fewest detected transcribed regions [123]. Whole blood seems to be a tissue type with less disease-specificity. However, it is also the most accessible biospecimen in clinical practice and could thus obtain sufficient test power with a large sample size. The differentially expressed gene is most likely to have the potential to be a biomarker and could be extensively used in clinical practice to help earlier diagnosis and disease classification.

Among seven TWASs associated with CD, five TWASs selected whole blood as targeted tissue. And among total 144 susceptibility genes in whole blood related with CD, *ATG16L1* [12, 13], Caspase recruitment domain 9 (*CARD*9) [12, 14], *ERAP2* [12, 13], *MICB* [12, 14], *NOD2* [10, 12, 13], *SLC22A5* [12, 14], and Tumor necrosis factor superfamily 15 (*TNFSF15*) [9, 10, 12] were overlapped between TWASs. *ATG16L1*, *ERAP2*, *MICB* and *SLC22A5* were overlapped with intestinal and discussed in the above.

Among the over 40 risk loci associated with CD identified to date, polymorphisms in *NOD2* account for the largest proportion of the genetic risk for this disease [124]. Experiments have demonstrated that NOD2 recognizes bacterial muramyl dipeptides and recruits ATG16L1 to bacterial entry sites on the plasma membrane, further regulating the intestinal barrier function and limiting transcellular permeability and bacterial translocation [125, 126]. However, the differential expression of *NOD2* in circulatory—but not intestinal—tissues is puzzling. NOD2 is widely expressed in macrophages and dendritic cells but to a lesser extent in intestinal epithelial cells [127] and T cells [128], which may explain this phenomenon. In addition, NOD2 can act as a viral sensor protein and is activated by the orally bioavailable dinucleotide SB 9200 [129], but whether it is effective against CD is unknown.

*CARD9* was a member of CARD family and an adaptor molecule predominantly expressed in lymphoid tissues and immune cells. The expression of *CARD9* was observed significant reduced in CD patients compared with healthy controls [130]. *CARD9* is a central signaling molecule in the innate immune via mediating NF-κB signaling and against fungi, bacteria, virus and mycobacteria [131–134], which is closely related to the pathophysiological of CD development.

*TNFSF15* is a Th-1-polarized cytokine that participates in systemic inflammatory responses and functions in regulating immune cells, inducing apoptosis, inducing inflammation, and inhibiting tumorigenesis, which suggests that *TNFSF15* is possibly a susceptibility gene in the blood. However, two studies of IBD observed that TNFSF15 was overexpressed in colonic tissues [135, 136]. The protective effect of *TNFSF15* polymorphisms on CD has been reviewed elsewhere [99, 137]. Notably, TNFSF15 was only screened in two Asian populations, Japanese and Korean, and was not found in other populations, and it can be tentatively speculated that the association of TNFSF15 with CD is stronger in Asian than in Western populations. Many previous studies have supported this hypothesis. A Japanese study reported a trend for a positive association between *TNFSF15* SNPs and the risk of anal lesions in CD [138]. Similar results were obtained in Chinese [139] and Korean population s[140]. In European population, a protective effect of *TNFSF15* was observed in CD but fail to define a clinical subgroup of CD patients specifically associated with *TNFSF15* [19]. A most recent study compared the susceptibility genes associated with IBD between two population from East Asian and European ancestries, respectively. In this study, researchers found that the genetic basis of CD appears to be more ancestral than that of UC due to the allele frequency of *NOD2* and the influence of *TNFSF15* [19]. And a meta-analysis also observed that East Asians gene have unique SNPs of *TNFSF15* associated with IBD [141]. And PF-06480605, an inhibitor of TNFSF15, has been developed and enter the Phase 2a clinical trial as a treatment strategy of UC patients (NCT02840721, Table 2).

The rough GO pathways enriched in whole blood mainly involves in the regulation of responses to biotic stimulus, including the regulation of defends system and the activation of immune system (Fig. 3D). This observation may suggest that the activity of immune cells and the process of inflammatory response in the whole blood may be affected by genetic background and reflect the disease status of CD.

## Future perspectives

**Fig. 4:**
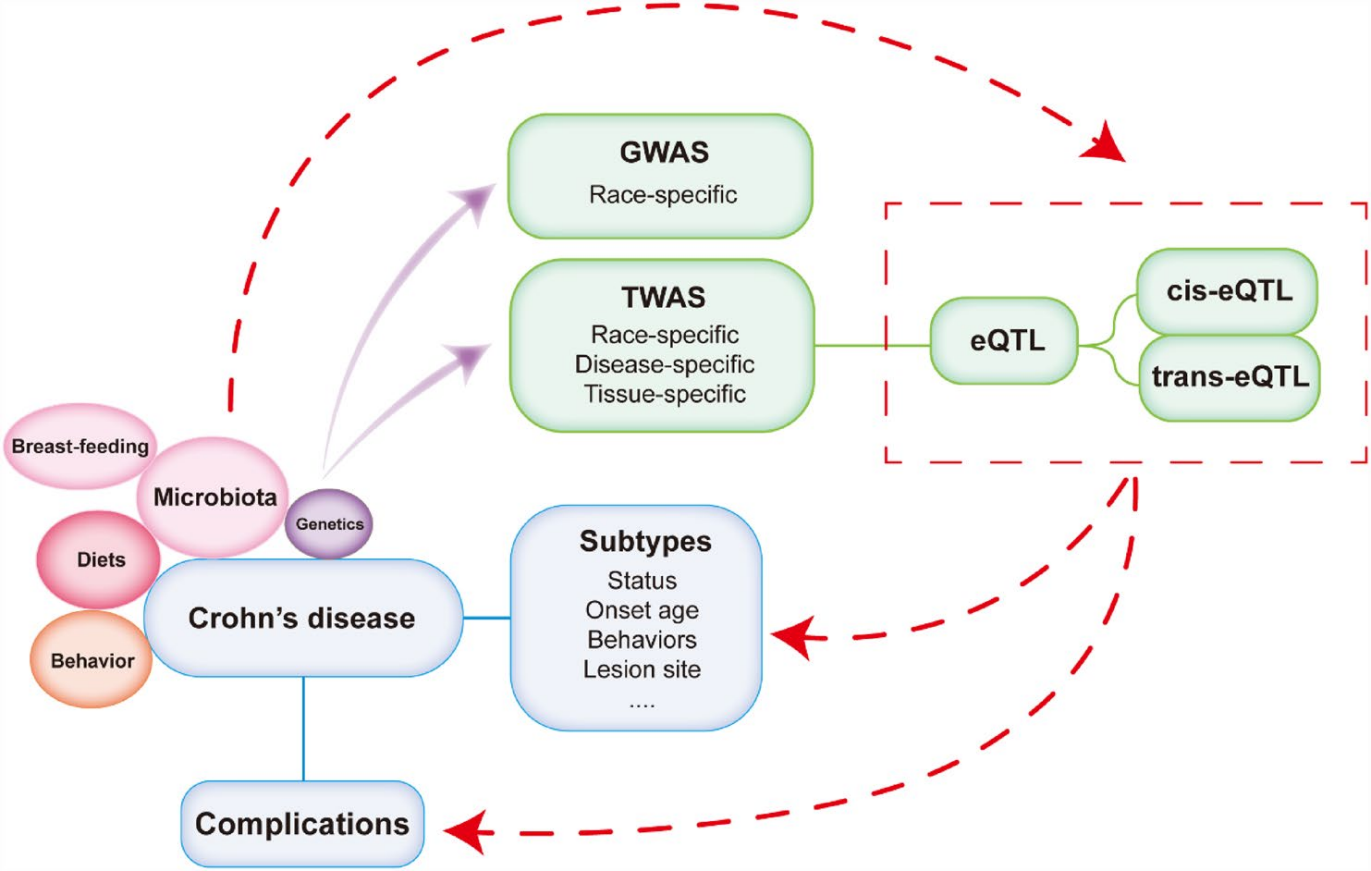
Overview of future research direction of TWAS in CD. GWAS, genome-wide association studies; TWAS, transcriptome-wide association studies; eQTL, expression quantitative trait loci

In this review, we summarized the susceptibility genes and enriched pathways associated with CD found in TWAS. Most susceptibility genes replicated between different tissues can only be observed in the same TWAS. For a fixed tissue type, susceptibility genes were rarely replicated in different TWAS. For instance, Uellendahl et al. identified *ATG16L1* as a susceptibility gene in almost all tissue types [13] but absent in Cheng’s multi-tissue TWAS [14]. Similarly, *MICB* was significantly associated with CD in Cheng’s study [14] but was absent in the other TWAS [9, 11–13]. In this section, we discuss the reasons for these issues, strategies for solving them, and future directions, as shown in Fig. 4.

Epidemiologic studies have reported that the incidence of IBD has now leveled off in developed areas but continues to increase in developing regions [142]. This phenomenon suggests that CD is more likely to result from the interaction of dietary behaviors and environmental factors with host immune mechanisms. The onset of CD can even be traced back to infancy; breast milk, containing oligosaccharides, contributes to the establishment of intestinal flora in infants and has a longer duration of benefit in inhibiting the adhesion of enteropathogenic bacteria and protecting against the development of the disease [142]. A recent study reported a transcriptome-wide association with gut microbiota [97]. Since numerous studies have demonstrated that the gut microbiota has a close relationship with CD pathology, the TWAS in the gut microbiota may provide new insights into investigating novel pathological mechanisms. This new gut microbiota-based tool may be influenced by diet and medication use, and its applicability to CD research remains to be demonstrated. Thus, broader exploration is needed, and the results should be interpreted with caution. Although over 200 genetic loci associated with IBD have been identified by GWAS, these variants can only explain a small proportion of the heritability of IBD (approximately 26% for CD and 19% for UC) [143]. No genetic markers have been reported to be predictive of complications [37, 144]. Considering that total expression is affected by genetic and environmental factors and that predicted expression in TWAS is only a part of the total expression, gene expression data assessed by genotype data and eQTLs have strong limitations and biases. The predicted expression in TWAS was generally slightly higher than the total expression correlations. The analysis of the correlation between predicted expression and CD may result in the significance of non-causal genes over causal genes due to linkage disequilibrium [32]. When a large proportion of genes are in linkage disequilibrium, the linkage disequilibrium region may also contain causal associations not related to the gene set. Even if there is no causal relationship between the gene set and the phenotype, it can still exhibit a significantly high rate [145]. Therefore, more work is needed to understand the genetic structure of CD.

Before the emergence of GWAS, most genotypic-phenotypic associations failed to replicate owing to small sample sizes, improper reliance on standard significance thresholds, failure to account for associations with low prior probabilities, and failure to assess the same SNPs across studies [1, 146]. We must acknowledge that the replication of TWAS results is not easy. The following reasons make the replication for CD TWAS even harder. (1) As a complex disease, the subtypes of CD patients, such as age of onset, lesion site, disease behaviors, disease process (active/remission), and medication used (hormone/biologicals), have different expression profile [42, 147], which should be included in GWAS datasets. Due to the lack of detailed information on large-scale genotype data sets, few studies have explored the relationship between predicted expression data and CD subtypes. This critically restricts further exploration of genetic factors in CD pathology. As a progressive disease, the onset time of symptoms and occurrence of complications are also important outcomes. (2) The establishment of eQTLs, including the sample size, ethnic consistency with the GWAS population, and different control populations, will also have a great impact on the TWAS results. (3) The rapid improvement and update of TWAS methods also reduce the reproducibility of TWAS results. Therefore, replication of these findings still needs a good deal of work in the future.

Disease behavior of CD changes over time, and patients with inflammation as the main presenting behavior at diagnosis are highly likely to develop fistulas or stricture complications within 20 years [148]. Prolonged inflammatory responses during clinical remission can lead to complications (strictures, fistulas, and abscesses) and progressive intestinal damage [149]. The Montreal classification considers in detail whether a prescribed time point should be given before disease behavior classification [5]. The homogeneity of participants and tissue type could effectively ensure the credibility of the results. Even compared with healthy people, the heterogeneity within CD patients can cause a large bias. Most of the populations in the available studies were heterogeneous, including patients with various lesion sites or different disease states (active or remission). There are also other kinds of population heterogeneity. For instance, Gettler et al. selected a TWAS population from a RISK cohort that recruited children and adolescents under 17 years of age [11, 150]. The susceptibility genes in this study may indicate a different pathology compared to that in the adult study. This Japanese study investigated differentially expressed genes between patients diagnosed with active CD and active UC, which may reflect different pathological processes in CD patients and healthy controls [9]. The heterogeneity of TWAS results in different tissue types is discussed at length above. Two IBD fine mapping studies published in 2017 found less than 30% concordance between eQTL and GWAS in identifying key genes [151, 152]. One study suggested a more significant overlap between eQTL and methylation QTL, and both studies suggested that related effects may be specific to cell type or disease status [151, 152]. During the past 100 years, the incidence of inflammatory bowel disease has sharply risen, then plateaued in the western world, whereas countries outside the western world seem to be in the first stage of this sequence [142]. The fact that CD patients are often treated for life and tend to concentrated in a few well-known treatment centers, it is feasible to obtain sufficient numbers of patients and biological samples through recruitment in well-known treatment centers. And a certain tissue type within the same study design is recommended to be consistent, allowing for increased sample size on a limited budget. In the future, larger-scale, cell/tissue-specific, and status-specific studies will be vital to resolve this problem. With the advancement of technology, single-cell RNA sequencing and single-cell TWAS have already emerged, substantially improving the homogeneity of samples and further facilitating targeted interpretations of TWAS outcomes and disease mechanisms for individual cell types or specific disease states [153].

Among the most frequently mentioned TWAS genes, such as *ATGL16L1*, *NOD2* and *IL23R*, were most reported by coding risk variants in GWAS studies instead of replicating the results of RNA-seq. Among the seven studies, only one TWAS provided the gene list associated with CD obtained by RNA-seq data [12]. Of the 95 associated genes screened by TWAS and the 35 associated genes screened by RNA-seq, only two genes (RPL9 and STMN3) overlapped. Since the most of RNA-seq data involved in the other six TWAS were either not associated with CD or did not include appropriate cases and controls (HC only, CD only, CD and UC), we found another two well-designed studies for comparison. Two Asina studies identified differentially expressed genes by RNA-seq in CD patients [154, 155]. Unfortunately, there was no susceptibility gene overlap between RNA-seq results and TWAS results neither in the ileum or colon. And the susceptibility genes screened by TWAS was less overlapped with the results of RNA-seq. There are several reasons for this phenomenon: (1) According to the distance of gene effect, eQTL includes cis-eQTLs (local) and trans-eQTLs (distal) [156]. A previous study observed that pervasive cis-eQTLs affect the majority of human genes (~ 75%) [157, 158], but a large twin study claimed that only 10% of the variation in gene expression was explained by cis-eQTL [123]. However, cis-eQTLs remain the only reliable tool in the TWAS method [32], which was limited in assessing the allele-specific expression [159]. (2) Stretch enhancers are large chromatin-defined regulatory elements that regulate the expression of cell type-specific genes and are enriched in disease-associated genetic variants in disease-associated cell types. However, eQTL effect sizes for stretch enhancers may be smaller than for ubiquitous promoter regions, which may lead to prediction bias [34]. (3) The pleiotropy, including horizontal pleiotropy and vertical pleiotropy, is widely existed in genome but the exact extent is still unknown [160]. Most of the genes may be indirect causative genes for complex traits, and some of the GWAS gene expression predicted by eqtl may be amplified due to horizontal pleiotropy [35]. (4) Gene expression may be affected by heritable epigenetic variation, small signaling molecules or other environment factors [161]. For example, *NOD2* expression could be induced by bacterial lipopolysaccharide, short-chain fatty acids, hormonal vitamin D, and TNF-α [162], which make it harder to predict the real expression levels. Although rarely reported in RNA-seq studies, these gene expressions are involved in mucosal immunity as previous reported [99, 162, 163]. This suggests that RNA-seq and TWAS may have complementary roles in explaining genetic associations of complex traits.

Counterintuitively, a susceptibility gene, such as *NOD2*, identified in a tissue type is not always consistent with its function. This observation raises the question of whether differential expression results obtained by eQTLs can explain causal associations, and a growing body of data has raised this question. As Wainberg et al. pointed out, the TWAS method is merely a statistical test to predict expression and disease risk from genetic evidence, which can be used to screen candidate disease-causing genes but does not guarantee causality [32].

In conclusion, the following three considerations might benefit future TWAS for CD, facilitating a more rational study design. (1) Despite its generic nature, we require GWAS data from different countries and disease states with large sample sizes. (2) The demand for a comprehensive classification, including race, tissue, lesion site, status, and progressive time points, is increasing with the accumulation of eQTL data. (3) Transcriptome-wide data combined with new technologies, such as single-cell approaches, will provide novel insights into the pathological mechanisms of CD and progress in TWAS. (4) In future TWAS, the results and data of intermediate processes should also be provided to facilitate the integration of data from multiple studies, dig deeper into genetic information, and provide more predictions for drug discovery.

### Supplementary Information

**Additional file 1.** The list of susceptibility genes in various tissue types screened out by different TWAS methods.

**Additional file 2.** All susceptibility genes associated with CD in various tissue types screened by TWAS.

**Additional file 3.** The total significant results of GO functional analyses. 

## Data Availability

Data sharing is not applicable to this article as no datasets were generated or analysed during the current study.
